# Single Canonical Model of Reflexive Memory and Spatial Attention

**DOI:** 10.1038/srep15604

**Published:** 2015-10-23

**Authors:** Saumil S. Patel, Stuart Red, Eric Lin, Anne B. Sereno

**Affiliations:** 1 Department of Neuroscience, Baylor College of Medicine, Houston, TX-77030; 2 Department of Neurobiology and Anatomy,University of Texas Medical School at Houston, Houston, TX-77030.

## Abstract

Many neurons in the dorsal and ventral visual stream have the property that after a brief visual stimulus presentation in their receptive field, the spiking activity in these neurons persists above their baseline levels for several seconds. This maintained activity is not always correlated with the monkey’s task and its origin is unknown. We have previously proposed a simple neural network model, based on shape selective neurons in monkey lateral intraparietal cortex, which predicts the valence and time course of reflexive (bottom-up) spatial attention. In the same simple model, we demonstrate here that passive maintained activity or short-term memory of specific visual events can result without need for an external or top-down modulatory signal. Mutual inhibition and neuronal adaptation play distinct roles in reflexive attention and memory. This modest 4-cell model provides the first simple and unified physiologically plausible mechanism of reflexive spatial attention and passive short-term memory processes.

Attention and memory are arguably the two most fundamental cognitive functions performed by the brain. Although there has been much research focused on varieties of attention and memory, much less is known about the nature of the relationship or interaction between these constructs (see^1^ for recent review). One important question is whether spatial attention and spatial short term or working memory share any common neural substrate. Whereas some results argue against a common neural substrate for working memory and attention^2^,^3^,^4^, many theoretical and empirical studies suggest a close relationship between attention and working memory in a variety of tasks^5^,^6^,^7^,^8^,^9^,^10^,^11^,^12^,^13^,^14^,^15^,^16^,^17^,^18^,^19^. Without clear well-defined mechanisms, such a question is difficult to test, much less resolve^20^.

Persistent activity during the delay period of delayed response tasks has long been proposed to be the neural correlate of short-term memory^21^,^22^ or long-term memory^23^ or associations^24^,^25^,^26^,^27^,^28^. Many laboratories have documented that neurons in the dorsal stream (e.g., lateral intraparietal area or LIP, prefrontal cortex) and ventral stream (e.g., infero-temporal or IT) exhibit stable persistent activity after a brief presentation of a visual stimulus^22^,^29^,^30^,^31^,^32^,^33^,^34^,^35^. Some studies using long-term visual association paradigms (e.g., fixed temporal order of stimuli across trials) have even demonstrated persistent activity during intertrial intervals^28^, whereas other studies using a delayed paired association task have shown active maintenance of internal representations during the delay period following presentation of intervening distractors^27^. Modeling studies of maintained activity in cortical neurons have suggested that it may be a result of equilibrium points of neural network dynamics in recurrent networks^28^,^36^,^37^,^38^,^39^,^40^,^41^,^42^,^43^,^44^,^45^,^46^,^47^,^48^,^49^,^50^,^51^,^52^,^53^,^54^,^55^,^56^,^57^,^58^,^59^,^60^,^61^,^62^.

A key distinction that is often overlooked in both attention and memory research is the difference between voluntary (top-down) and reflexive (bottom-up) processes^29^,^63^,^64^. Such a distinction is likely to relate to different physiological mechanisms, and thus failure to distinguish between fundamentally different processes may lead to confusions. In support of the importance of such distinctions, one recent study^27^ that distinguished between a more passive (or “cue holding”) versus active (or “target-recall”) delay activity demonstrated that intervening distractors in a modified paired association task interfered with cue-holding (bottom-up, passive) delay activity but not the target-recall (top-down, active) delay activity. Although, given the important role the cue plays with respect to predicting the target, some may argue that any cue-holding activity may be more top down than a cue stimulus with no predictive value. Thus, a second important question is to what extent does the persistent activity observed in dorsal- and ventral-stream neurons represents a more passive memory trace (or kind of priming) resulting from visual stimulation regardless of the specific task at hand, or conversely a more active modulation or memory trace that represents what the animal is actively remembering in order to correctly perform the task. Many studies suggest that the persistent activity represents an active or voluntary mnemonic process (e.g.,^65^,^66^). However, these studies have not controlled for passive (or reflexive) memory traces. This question is difficult to answer from studies using delayed response tasks or delayed paired associate tasks because there is typically no control for the visual stimulation, per se. To address the question of passive versus active processes, Sereno and Amador^29^ recorded single neuron activity in LIP when the monkey was performing two delayed match-to-sample (DMTS) tasks that were randomly interleaved. The two tasks (a shape memory task and a location memory task) were designed to use identical visual stimulation (see Fig. 1 in^29^) and only differed in the information that the monkey had to remember. In the shape DMTS task, the monkey had to remember the shape of the sample stimulus whereas in the location memory task the monkey had to remember the location of the sample stimulus. In the shape memory task, following a delay, a test display was presented and the monkey had to saccade to the location of the stimulus whose *shape* matched the shape of the remembered stimulus. In the location DMTS task, the animal had to saccade to the remembered *location* of the sample stimulus regardless of which shape appeared at that location in the test display. Note that in both tasks, the response was a localization response. The neural recordings showed that many cells in LIP exhibited spatial and shape selective persistent activity. More importantly, the neural recordings showed evidence for both passive and active memory traces. First, many spatial or shape selective cells showed selectivity related to the sample stimulus but their persistent activity was unmodulated by the memory task that the monkey was performing, suggesting that much persistent activity in LIP represents a more passive memory trace (such as a shape or spatial priming). Second, a smaller percentage of LIP cells exhibited persistent activity that *was* modulated by the memory task. Together, these finding suggest that there exist populations of LIP cells that mediate both passive (reflexive) and more active (voluntary) memory processes.

We previously developed a model of *reflexive spatial* attention based on physiological properties of neurons in LIP^63^,^67^ which accounted for fundamental aspects of reflexive spatial attention: namely, the facilitatory and inhibitory cueing effects dependent on the relative location and timing of cue and target. In addition, the model made specific unexpected behavioral predictions about how the shape of the cue and target would influence the reflexive spatial attention time course, predictions that were verified in experiments conducted with human subjects^67^,^68^. Surprisingly, the neurons in this model of reflexive spatial attention also displayed stable persistent activity after brief visual stimulation. It is important to note that in a reflexive attention paradigm, there are two stimuli presented on each trial much like a delayed match-to-sample or delayed paired associate task, however there is no short-term association (e.g., delayed match to sample) or long-term association either between the cue and upcoming target or in the temporal order of stimuli across trials. That is, the cue is task irrelevant and unrelated to (unpredictive of) the upcoming target; further the task is focused entirely on the target (in this example, localizing the target). We suggest here that this persistent activity that appeared in the model may be the neural correlate of a passive spatial memory, similar to the activity found in many LIP neurons, and that the model proposed here is a canonical model that can explain passive spatial memory as well as reflexive spatial attention. In this study, we examine in detail (1) how persistent activity arises in this reflexive-spatial-attention neuronal network, (2) what conditions are necessary, (3) what conditions modulate this persistent activity, and (4) how different parameters of the model similarly or differentially affect attention and mnemonic processes.

## Model and Modeling Results

**Figure 1:**
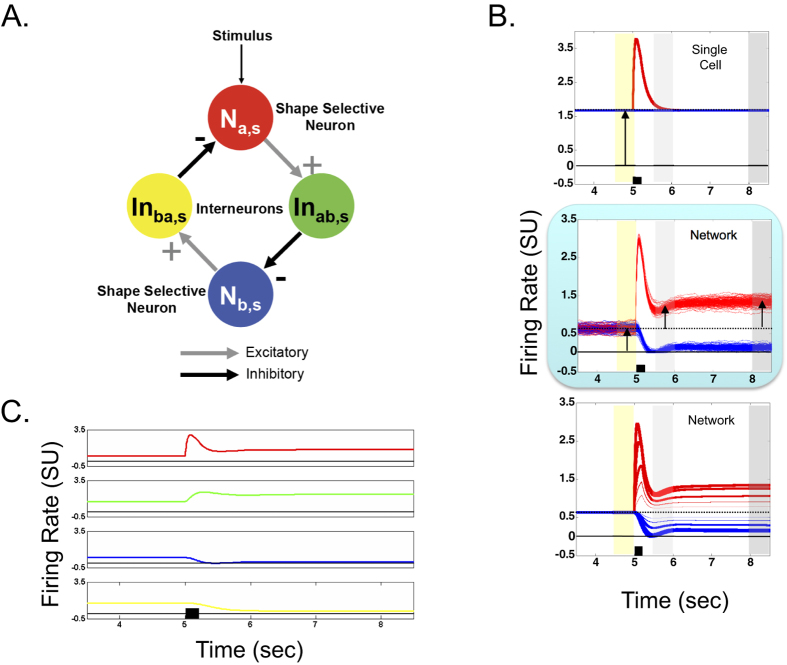
(**A**) module from the neurophysiologically based model of reflexive spatial attention^67^ and example simulation results. (**A**) This network encodes a particular spatial location ‘s’ and consists of 2 shape selective neurons (red prefers shape ‘a’ and blue prefers shape ‘b’) and two interneurons (green and yellow). Gray (black) arrows indicate excitatory (inhibitory) connections. (**B**) Simulation results of the network module shown in (**A**). The colored traces represent the responses of the corresponding shape selective neurons. The firing rates in this and all other figures are plotted in arbitrary simulation units (SU). The stimulus pulse duration is indicated by the short black horizontal bar immediately above the x-axis. The yellow, light gray and dark gray rectangles represent the time interval over which the firing rates were averaged to yield a single value of baseline activity, short term maintained activity and longer term maintained activity, respectively. Maintained activity is defined as the average activity over a time period (short term or longer term) minus the baseline activity. The arrow during the yellow baseline activity interval indicates the non-zero baseline firing rate of both neurons. The black dotted line represents baseline activity and the black solid line, a firing rate of zero. *Top panel*- simulation firing rates (or activities) of the two shape selective model neurons when they are in isolation (inhibitory inputs are removed). The red neuron is stimulated by a 200 msec pulse at 5 sec and shows no persistent activity. Note, the blue neuron trace is overlying the dotted baseline activity. *Middle panel*- simulation firing rates (or activities) of the two shape selective model neurons of intact network module with added uniform distribution random noise. The firing rate of the red neuron increases rapidly and then gradually decays to a persistent level (under the light and dark gray rectangles; arrows in these gray rectangles represent maintained activity) that is higher than the baseline activity. *Bottom panel*- simulation firing rates (or activities) of the two shape selective model neurons of intact network module for different strengths of the input. (**C**) Responses of all the model neurons to a 200 ms stimulation of the red shape selective neuron.

The same model that was used to account for reflexive spatial attention^67^ is used in this article and therefore is briefly described below. The model in Patel *et al*.^67^ considered two spatial locations and therefore consisted of two identical neural subnetworks each of which represented the processing at one spatial location. Here, we have considered the processing at a single spatial location (location “s”) and therefore the model in this study (shown in Fig. 1a) consists of just one neural subnetwork from Patel *et al*.^67^. The mathematical description and parameters of the model used here are in the supplementary materials (see Mathematical Description of the Model and Table S1). The two parameters in bold have values that are changed from the values used in Patel *et al*.^67^ For comparison, the values of these parameters used in Patel *et al*.^67^ are in parentheses. These parameters were changed to clearly illustrate the baseline and maintained activities but qualitatively similar results for the memory model reported here are obtained with the original values. Further, the results of reflexive spatial attention with the changed (bolded) values (see Figure S2 in supplementary material for attentional results with changed parameter values) are also qualitatively similar to those reported in Patel *et al*.^67^.

The network shown in Fig. 1A encodes for a particular spatial location ‘s’ and consists of two shape selective neurons: a red one that prefers shape ‘a’ and a blue one that prefers shape ‘b’. There are also two inhibitory interneurons, green and yellow. Gray arrows indicate excitatory connections and black arrows indicate inhibitory connections. The red neuron excites the green interneuron, which in turn, inhibits the blue neuron. Similarly, the blue neuron excites the yellow interneuron, which in turn, inhibits the red neuron. This pattern of connectivity implements mutual inhibition among shape selective neurons. Mutual inhibition has been previously hypothesized to be responsible for maintained activity and decision-making by prefrontal cortical neurons^62^.

To preview the ability of this network to exhibit a passive memory trace, in a computer simulation, the red neuron was stimulated by a 200 millisecond pulse, signifying a 200 ms presentation of the shape ‘a’. The top panel in Fig. 1B shows the firing rates (or activities) of the two shape selective neurons when they are in isolation (inhibitory inputs are removed) whereas the middle panel shows the firing rates of the same neurons when they are in the network. The non-zero baseline activity in red and blue neurons is evident in both panels (time period marked with the yellow rectangle). Also, in both panels, the firing rate or output of the red neuron increases rapidly after stimulus presentation (indicated by the short black horizontal bar immediately above the x-axis) and then gradually decays. However, in the network (middle panel) compared to isolation (top panel), even in the presence of noise (see membrane activity equations in supplementary material), the activity of the red neuron is substantially higher than the baseline activity immediately following stimulus presentation (short-term maintained activity during time period marked with the light gray rectangle; maintained activity is defined as the activity above the baseline activity). Further, at longer intervals (time period marked with the dark gray rectangle), the activity is identical to the baseline activity when neurons are in isolation (top panel) but is substantially higher than the baseline activity in the network (middle panel). The magnitude of maintained activity is directly related to the strength of the response to the stimulus (bottom panel), which could be thought of as the stimulus selectivity of the cell. To demonstrate that the model parameters utilized here generate dynamic responses consistent with those of LIP neurons, the normalized response of a model shape selective cell and a shape selective cell in LIP are superimposed in Figure S3A in the supplementary material. The only change made to the parameters used for simulations in this paper, and listed in the supplementary material, was that *I*_*exc*_: on was changed from 10 to 4. Additionally, Figure S3B in supplementary material shows that the model neuron used in Patel *et al*.^67^ also demonstrates activity that is elevated above the baseline activity.

In summary, when the model neuron is briefly stimulated, activity, which is directly related to the stimulus response strength, is maintained at an elevated level compared to the baseline both immediately after the stimulus is removed as well as after a longer duration. Importantly, the dynamic response of the shape selective model neuron is consistent with that of a shape selective neuron in LIP (see Figure S3A). Further, the maintained activity in the model neuron is not a property of a single neuron but arises as a result of the network connectivity. This maintained activity exists without any top-down modulation in the model and thus may form the basis for a passive memory trace discussed earlier.

To generate meaningful predictions, it is important to understand what in the model causes maintained activity in the shape selective neuron. Mutually inhibitory networks with linear neurons have been well studied by^58^; however, the neurons used in our model are non-linear. That is, the model neuron’s membrane potential changes non-linearly with respect to its inputs (see equations in the Mathematical Description of the Model section and Table S1 in the Supplementary Material). Thus, a closed form solution does not exist for the set of ordinary differential equations that comprise the model. Therefore the model has to be examined using numerical simulations. To gain some intuition about the mechanism that causes maintained activity, we first examine the firing rate pattern in all four neurons (in network) in response to the same stimulation that was used for the middle panel of Fig. 1B (i.e., 200 ms pulse to the red neuron). Figure 1C shows that prior to any stimulation, all the neurons have a non-zero steady firing rate. These traces provide some intuition regarding the mechanism behind maintained activity in the shape selective neurons. In response to stimulation of the red shape selective neuron, this neuron’s activity quickly rises which in turn causes the activity of the green interneuron to rise. The rise in activity of the green interneuron causes the blue shape selective neuron to gradually reduce its firing rate virtually to zero. This in turn gradually reduces the firing rate of the yellow interneuron, which in turn will reduce the inhibition of the red shape selective neuron. Thus, for each shape selective neuron, a positive feedback loop is formed. With the proper neuronal and network parameters, the reduction of inhibition (or “disinhibition”) of the red shape selective neuron will cause its firing rate to remain elevated up to many seconds after the initial stimulation.

This mechanism of maintained activity resembles previously proposed memory mechanisms that were based on positive feedback or reverberations in a network of neurons^36^,^37^,^38^,^39^,^40^,^41^,^42^,^43^,^44^,^45^,^46^,^47^,^48^,^49^,^50^,^51^,^52^,^53^,^54^,^55^,^56^,^57^,^58^,^59^,^60^,^61^,^62^. However, systems with positive feedback can exhibit instability. Thus, below we discuss how various neuronal parameters modulate the maintained activity and affect the stability of maintained activity in the model. For the simulation results discussed in each of the following sub-sections, only the parameter under investigation was varied, while the rest of the parameters were identical to those used for Fig. 1B (middle panel, network configuration).

### Relationship between mutual inhibition and maintained activity

**Figure 2:**
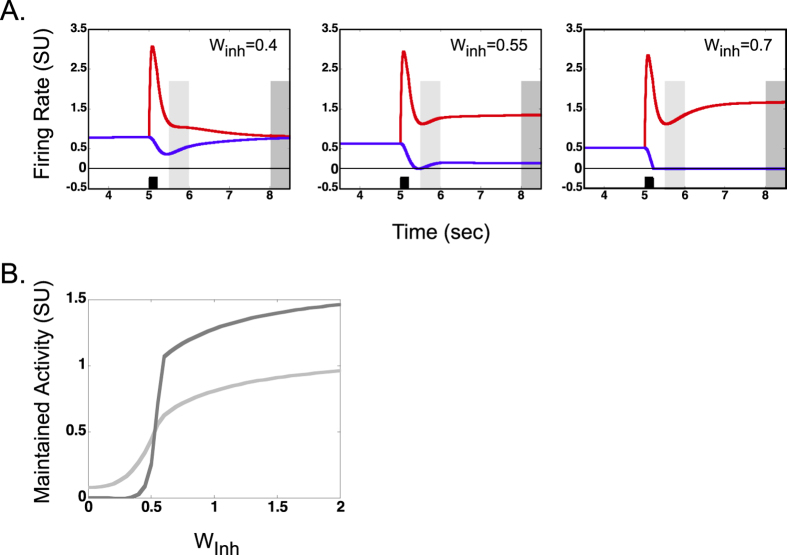
Relationship between mutual inhibition and maintained activity. (**A**) Example firing rates of shape selective neurons for different strengths of the inhibitory synaptic weights (W_inh_) in the model. The red neuron was stimulated by a 200 msec pulse at 5 sec. (**B**) The strengths of short (light gray curve) and longer term maintained activity (dark gray curve) are shown for a range of inhibitory synaptic weights. Strength of maintained activity was measured during the short (light gray) and longer (dark gray) time intervals (illustrated in Fig. 2A).

As noted earlier, disinhibition of a stimulated shape selective neuron causes the neuron to maintain its firing rate above its baseline. Hence we manipulated the strength of the inhibitory connections and examine how it affects the maintained activity. Figure 2A shows the responses of the red and blue shape selective neurons as their inhibitory synaptic weights (W_inh_) are varied (all other parameters remained same as those used for Fig. 1B’s middle panel, network configuration). Each panel in Fig. 2A shows the responses for a specific value of W_inh_ (increasing from left to right) with the middle panel being identical to that shown in Fig. 1B (middle panel, network configuration).

First, notice that for the left and middle panels, after the red neuron is stimulated, the blue neuron decreases its response but is not turned off for an extended period of time. On the other hand, in the right panel, the blue neuron stops firing shortly after the red neuron is stimulated and remains silent until the network is reset. The model operates in one regime for the left and middle panels and a second regime for the right panel.

In the first regime (single equilibrium regime; left and middle panels in Fig. 2A), for a given set of parameters, there is just one stable equilibrium point and this equilibrium point corresponds to the baseline activities in the model. Note that at equilibrium, the activities of all the neurons in the model reach steady-state. By stable equilibrium point we mean that in the absence of any stimulation or after any perturbation, the activities of the neurons would settle on this equilibrium point. Thus, any short-term maintained activity eventually decays back to the original baseline activities. The strength of the inhibitory synapse (W_inh_) along with other parameters determines the rate at which the maintained activity decays. For example, in the left panel (W_inh_ = 0.4), the decay is relatively rapid whereas in the middle panel (W_inh_ = 0.55), the decay is slow. It should be noted that the initial transient response to the stimulation of the red neuron is very similar in all the panels (compare peaks in all panels).

In the second regime (dual equilibrium regime; right panel in Fig. 2A), for a given set of parameters and highest inhibitory synaptic weights, there are two equilibrium points. One corresponds to the baseline activity in the model, but this is an “unstable” equilibrium point. By unstable equilibrium point, we mean that a small perturbation causes the neurons output to reach a second equilibrium point that is different from the initial equilibrium point. This second equilibrium point is stable and corresponds to the “maintained activity” in the model. These statements were confirmed: 1. empirically by noting that a small perturbation around the baseline activity of the red shape selective neuron caused its activity to change towards the second, stable, equilibrium point and 2. analytically by solving the constrained differential equations (constraint: all cells are active, so the firing rate non-linearity is eliminated) for a steady state and noting that the baseline activities actually correspond to an equilibrium point (even if unstable). The longer term “maintained activity” in the dual equilibrium regime is independent of the stimulus strength (i.e., selectivity, see dark gray line in Figure S1) and thus this regime is expected to be largely irrelevant for remembering a *particular* stimulus. However, it is possible that the stable equilibrium point in the dual equilibrium regime represents uncontrolled neuronal activity that occurs in epilepsy and other diseases that are associated with hyperactivity in the brain.

Figure 2B summarizes the short-term (light gray trace) and longer-term (dark gray trace) maintained activities for a range of inhibitory synaptic weight values. Strength of the short- and longer-term maintained activities were measured as average activity during their respective time intervals as illustrated by light and dark gray boxes, respectively, in Fig. 2A. The single and dual regimes described earlier are also seen in Fig. 2B. There is a sharp transition in the level of longer-term maintained activity (dark gray curve) around W_inh_ of 0.5 and somewhere between W_inh_ of 0.55 and 0.7, there is a transition from the single to the dual equilibrium regime. Note that the corners of the curves in Fig. 2B do not correspond to the regime transition points. The inhibitory weight corresponding to the transition point is difficult to determine analytically and therefore we have just provided a range based on our empirical analyses. Figure S1 illustrates that the maintained activity, when Winh = 0.7, is independent of the input level suggesting that the model is operating in the dual equilibrium regime for a large input range.

In summary, maintained activity for various lengths of time can exist in the same model^67^ that also demonstrates reflexive spatial attention. Further, different neuronal and network parameters allow the model to operate in different regimes that may have physiological relevance. Mutual inhibition in the network is one of the important parameters for duration and magnitude of maintained activity in the model as well as determinant of stable states of the model.

### Relationship between repetition suppression and maintained activity

Repetition suppression or reduced response to a repeated stimulus is a property of many dorsal^30^ and ventral^69^,^70^,^71^,^72^,^73^,^74^,^75^,^76^,^77^,^78^ stream neurons. Repetition suppression is also a property of the model neurons and was found to be critical for explaining spatial attentional cueing effects and their dependence on shapes of the cue and the target^67^. Here, we examine the effect of repetition suppression on maintained activity.

**Figure 3:**
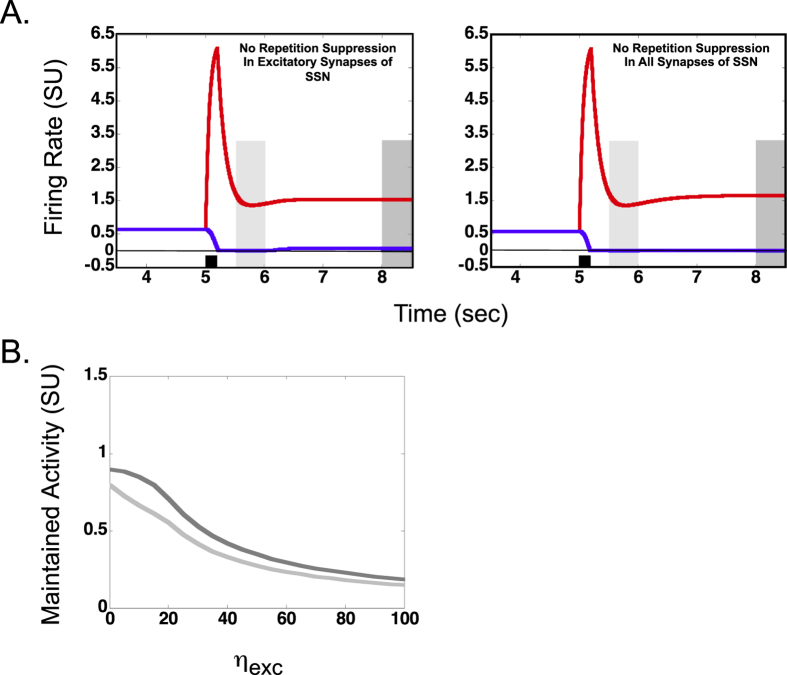
Relationship between repetition suppression and maintained activity. (**A**) Example firing rate of shape selective model neurons in a condition in which the repetition suppression was removed from excitatory synapses of shape selective neurons (left) and from both excitatory and inhibitory synapses (right). The red neuron was stimulated by a 200 msec pulse at 5 sec. Conventions used in panel (**A**) are consistent with Fig. 1B. (**B**) The strengths of short and longer term maintained activity are shown as a function of a parameter which determined the peak adaptive gain change in the excitatory synapse (η_exc_). Zero on the x-axis represents no adaptive gain control and thus no repetition suppression. Increasing value of η_exc_ represents increasing repetition suppression. Conventions used in panel (**B**) for short and longer term maintained activity (light and dark gray traces) are consistent with Fig. 2B.

Figure 3A shows the response of the red and blue shape selective neurons when the repetition suppression property is eliminated from excitatory synapses of both shape selective neurons (left panel) and from both excitatory and inhibitory synapses (right panel; the network otherwise under the same stimulation and parametric conditions as in middle panel of Fig. 1B, without the added noise).

As can be seen in the left panel in Fig. 3A, the elimination of the repetition suppression property in shape selective neurons does not eliminate the maintained activity in the red neuron. However, as seen in Fig. 3B, increased repetition suppression in shape selective neurons (larger values of η_exc_) decreased the magnitude of both the short- (light gray curve) and longer-term (dark gray curve) maintained activity. Thus it is clear that the repetition suppression property in shape selective neurons is not necessary for the existence of maintained activity but that the maintained activity is modulated by the magnitude of repetition suppression. The model operated in the single equilibrium regime for almost the entire range of η_exc_.

On the other hand, as shown in the right panel in Fig. 3A, elimination of repetition suppression property from both excitatory and inhibitory synapses in the model (η_exc_ and η_inh_ ) causes the model to operate in the dual equilibrium regime. Thus, the existence of repetition suppression in inhibitory cells in the model is necessary for the single equilibrium regime and hence, a critical determinant of the regime in which the model operates.

### Relationship between baseline and maintained activity

**Figure 4:**
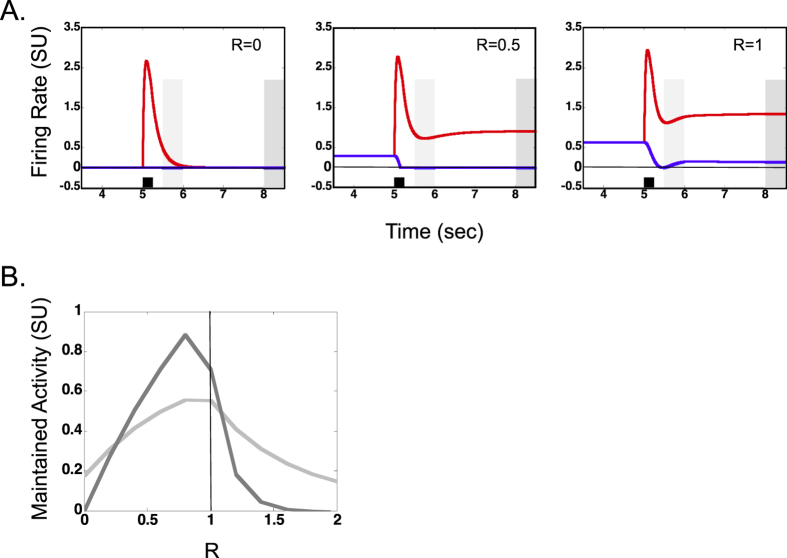
Relationship between baseline activity and maintained activity. (**A**) Example firing rates of shape selective neurons for different levels of baseline activity (determined by R) in the model. The red neuron was stimulated by a 200 msec pulse at 5 sec. Conventions used in panel (**A**) are consistent with Fig. 1B. (**B**). The strength of short- (light gray) and longer-term maintained activity (dark gray) is shown as a function of R in the model. Vertical line indicates transition from dual equilibrium regime (left side of line) to single equilibrium regime. Conventions used in panel (**B**) for short and longer term maintained activity (light and dark gray traces) are consistent with Fig. 2B.

Many dorsal and ventral stream neurons have spontaneous firing in the absence of visual stimulation (e.g. see^30^). Figure 4 shows the responses of the red and blue shape selective neurons as the baseline activity (parameter R) was varied in the model. Each panel in Fig. 4 shows the responses for a specific value of R (increasing from left to right), with the right panel being identical to the stimulation and network configuration shown in middle panel of Fig. 1B. As indicated by the left panel, there was no longer term maintained activity without baseline activity. For the middle panel, the model was operating in the dual equilibrium regime whereas for the right panel it was operating in the single equilibrium regime. Thus, there is a minimum level of baseline activity necessary for model operation to be in the single equilibrium regime and this minimum level occurs when R is somewhere between 0.5 and 1. The summary of the relationship between baseline activity and short and longer-term maintained activity is shown in Fig. 4B. Again note that the curve representing longer term maintained activity (dark gray) passes through the origin indicating that there was no longer-term maintained activity without baseline activity. The light gray curve indicates that there was short-term persistent activity without baseline activity but it decayed rapidly to zero (also see left panel in Fig. 4A). Thus baseline activity is important for existence of longer term maintained activity and a minimum level of baseline activity is necessary for model operation in the single equilibrium regime. In the single equilibrium regime, baseline activity is inversely related to maintained activity.

### Relationship between other parameters and maintained activity

Consistent with a biological neuron, each neuron in our model has a non-linear relationship between its membrane potential and its firing rate. This non-linear relationship is modeled by a linear above firing threshold (parameter 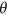) function. In the single equilibrium regime, longer-term maintained activity existed only for a certain range of firing rate thresholds (not illustrated) suggesting that the non-linear relationship between membrane potential and firing rate is necessary for longer-term maintained activity.

As in a biological neuron, the membrane potential of an isolated model neuron decays passively in the absence of external inputs. As expected (and therefore not illustrated), the longer term maintained activity decreased with increase in the passive decay constants (or equivalently, decrease in passive decay *time* constant) of either the shape selective neurons or interneurons. Depending on the passive decay constants of shape selective neurons and interneurons, the model either operated in single or dual equilibrium regime. Thus, as expected, the passive decay constants are also an important parameter for controlling the duration of maintained activity and stability in the model.

### Response to other input: voluntary control or new input

**Figure 5:**
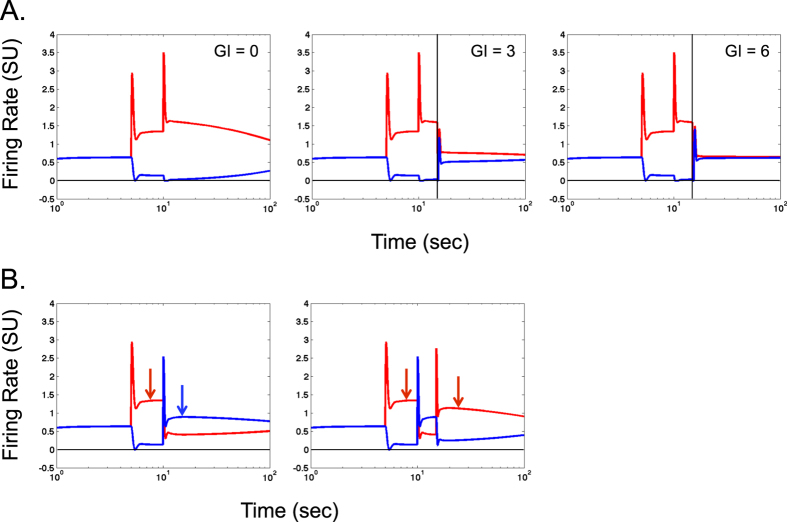
Reset of the passive memory trace and model’s response to novel input. (**A**) Example firing rates of shape selective neurons for increasing levels of gobal inhibition (GI) in the model. The red neuron was stimulated by two 200 msec pulses at 5 and 10 sec. All the cells were inhibited by a 200 msec pulse at 20 sec. Conventions used in panel (**A**) are consistent with Fig. 1B. (**B**) Example firing rates of shape selective neurons for a novel input. In the left panel, the red neuron was stimulated by a 200 msec pulse at 5 sec. The blue neuron was stimulated by a 200 msec pulse at 10 sec (novel input). Notice that the red neuron’s response drops below baseline after the activation of blue neuron. In the right panel, which has the same initial stimulation pattern as the left panel. The only difference is that the red neuron receives a second 200 msec pulse at 15 sec (also a novel input). Notice that the red neuron’s response starts from a level which is below the baseline. Conventions used in panel (**B**) are consistent with Fig. 1B.

In certain conditions, it may be important to suppress or counter passive maintained activity via voluntary control. It is also important to know how the shape selective neurons that have maintained activity respond to new sensory inputs. Figure 5A shows the response of red and blue shape selective neurons when the red cell is stimulated by two successive brief pulses (first input, shape a, followed by a new input, shape a again). Each stimulus was identical to that used for Fig. 1B and the model was operating in the single equilibrium regime. In addition, for middle and right panels in Fig. 5A, all the neurons in the model were inhibited at the 15 sec time instant (vertical line in middle and right panels) via an external signal representing voluntary control (global inhibition, GI). Such inhibition has been proposed previously to remove memory traces^37^. Each panel shows responses for a specific strength of external global inhibition. The panels in Fig. 5A show that 1. the transient response (or change in response) to a new second input is slightly smaller compared to that for the first input, each measured from the immediately preceding baseline activity (i.e., a repetition suppression^30^,^63^,^79^), 2. the activity in the red cell returns to baseline with increasing rate as the strength of global inhibition is increased and 3. the maintained activity for the new input (relative to original baseline) is higher than the maintained activity of the first input suggesting a form of *memory priming* if the first and second inputs have the same feature, in this case, shape^80^. Figure 5B shows the responses of red and blue shape selective neurons (with no global inhibition) when the new input is a different shape (hence delivered alternately to the two neurons). Both panels show that the maintained activity is lower (relative to original baseline) for the new input if the new input has a different shape than the previous input suggesting a form of *proactive memory interference*^81^ that is reflexive and specific to a spatial location (compare activities indicated by arrows in each panel). Further, the maintained activity of the old input is eliminated by the new input suggesting a form of memory erasure or masking. Such memory masking is expected from a mutually inhibitory network.

In summary, the canonical model of maintained activity and reflexive spatial attention can continue to respond normally to sensory signals and may also be reset in an accelerated manner under conditions of cognitive control. Further, the model exhibits aspects of memory priming, proactive memory interference and memory masking, processes that, in our model, can also be attributable to simple bottom-up processes.

## Discussion

### Single model for reflexive attention and memory

A modest model consisting of two shape selective neurons and two inhibitory interneurons previously explained reflexive spatial cueing effects (both facilitation and inhibition of return) as well as predicted novel and peculiar effects of shape on spatial attention. In this study, we show that these shape selective neurons in the same model also exhibit maintained activity after a brief visual stimulation similar to passive maintained activity previously reported in monkey physiology^22^,^29^,^30^,^31^,^32^,^33^,^34^,^35^. The maintained activity is also robust and is proportional to the response strength of these neurons (Fig. 1). The maintained activity in the model is not a property of any individual shape selective neuron but arises within a network of mutually inhibited shape selective neurons. More importantly, maintained activity occurs in these neurons without any top-down modulation. We are unaware of any other model that demonstrates this property together with the ability to explain the bimodal behavioral effects of reflexive spatial attention. Thus this model may represent a canonical circuit that can simply, as a result of its properties and configuration, account for multiple cognitive functions automatically.

### Clarification and cautionary note

By no means do we want to imply that reflexive spatial attention and passive memory are mediated by four cells but rather we present and demonstrate that a simple unified model has a rich set of dynamic properties that can help us understand various behavioral observations related to these two “cognitive” processes. Most importantly, the model demonstrates how two different cognitive processes may share the same “network” and yet have different parameters and properties that in some cases independently and/or differentially affect and determine these two behaviors.

### Necessary and modulatory conditions for maintained activity

Mutual inhibition and repetition suppression were key model properties to account for the reflexive spatial attention data and therefore we investigated their roles in the existence of maintained activity and the stability of the model. We found that the strength of mutual inhibition controlled the duration of maintained activity and the stability in the model. For the more useful single equilibrium regime of model operation, all other parameters remaining fixed, the level of mutual inhibition had to be lower than a critical value (Fig. 2). Below this critical level of mutual inhibition, the duration and level of maintained activity decreased with decrease in strength of mutual inhibition. Note that the transient response remained similar in this single equilibrium regime as well as the dual equilibrium regime. Because the results of reflexive spatial attention are largely based on this transient response, the choice of the strength of mutual inhibition within a specific range does not alter the qualitative aspects of the results of reflexive spatial attention derived from the model (compare Figure S2A and S2C in supplementary material). Mutual inhibition within this network therefore appears to be necessary for both reflexive spatial attention and memory, however the strength of mutual inhibition specifically modulates the duration of the passive memory trace. Thus, the model suggests that changes in inhibition that may occur, for example in a disease process, could affect passive memory but not reflexive attention (i.e., appear as a distinction between passive memory and reflexive spatial attentional processes), even while both processes are sharing a common neural substrate.

Further, repetition suppression in excitatory synapses of shape selective model neurons, a commonly observed property of dorsal and ventral stream neurons^30^, did not affect the stability in the model nor was it necessary for the existence of maintained activity (Fig. 3A, left panel). The model remained in the single equilibrium regime of operation for the entire range of repetition suppression strengths tested in excitatory synapses. As expected, there was a reduction of maintained activity with increased strength of repetition suppression in excitatory synapses (Fig. 3B). However, repetition suppression in the inhibitory synapses is critical in determining the regime of model operation (compare Fig. 3A left and right panels). This observation suggests that robust memory circuits require repetition suppression in the inhibitory synapses and that excitatory and inhibitory neurons may have distinct biophysical properties and roles in cognition.

Baseline activity of a shape selective neuron, another commonly observed property of dorsal and ventral stream neurons^30^, was also found to influence the existence of maintained activity and stability of the model. There was no maintained activity without baseline activity (Fig. 4A). Further, a minimum level of baseline activity was necessary for single equilibrium regime operation of the model (Fig. 4A). In this regime of the model, the maintained activity decreased with increased baseline activity (Fig. 4B). Note that the transient response decreased only slightly with increased baseline activity and again the choice of baseline activity does not significantly alter the qualitative aspects of reflexive spatial attention results derived from the model (compare Figure S2A with S2B in supplementary material). Again, this is a distinction between passive memory and reflexive spatial attentional processes that may appear as a dissociation, despite both processes sharing a common neural substrate.

In the single equilibrium regime, the transition from elevated activity level back to baseline level can be facilitated by simultaneously and briefly inhibiting all the neurons in the model (Fig. 5A). This suggests a potential role in memory related functions for inhibitory interneurons (such as Martinoti cells) that are known to widely connect to both excitatory and inhibitory cortical cells^82^,^83^. In the dual equilibrium regime, such a transition is virtually impossible because the baseline level is an unstable equilibrium point. Although in both regimes, the shape selective neurons in the model respond to new visual stimulation in a similar manner. Thus the regime of operation is less critical for reflexive spatial attention and more critical for maintained activity. As noted earlier, in the single equilibrium regime, the model also exhibits a reflexive form of memory priming, proactive interference and memory masking (Fig. 5B).

### Reinterpretation of prior findings

Such an overt model may be useful to test and reinterpret other data as well. In a reflexive spatial attention study in the superior colliculus, Dorris *et al*.^79^ found evidence for inhibited neural responses to a repeated stimulus (and the magnitude of this suppressed response correlated with slowing of saccadic reaction times) but argued these neurons were not the site of inhibition because these neurons were, in fact, more active following the presentation of the first stimulus in their response field (i.e., maintained activity). Further, they demonstrated that these neurons had a higher level of excitability, producing shorter latency saccades with electrical microstimulation. Hence, they argued that the reduced activity to the second stimulus in the superior colliculus neurons reflects a signal reduction that has taken place upstream, perhaps in parietal cortex. We demonstrate here that this pattern of suppression for a repeated stimulus (see negative spatial cueing effects in supplementary material Figure S2; see also^67^,) and maintained activity between stimulus presentations does not require an upstream signal reduction. The model neurons used in our model inherently exhibit an adaptive gain property such that the gain is reduced with increase in baseline activity. Therefore, the observation by Dorris *et al*.^77^ of a reduced response to a second stimulus (in cued compared to uncued trial) despite a higher maintained activity due to first stimulus (in cued compared to uncued trial) is in perfect agreement with our model. Thus, an explicit neurophysiologically plausible model is useful not only to better understand mechanisms of attention and memory at a single cell level, but may also provide insight into deficits of attention and memory, and even suggest optimal interventions.

### Parallel and distributed processing of reflexive attention and memory

Several modeling studies have proposed that memories are stored as equilibrium points in a dynamical system^36^,^37^,^38^,^39^,^40^,^41^,^42^,^43^,^44^,^45^,^46^,^47^,^48^,^49^,^50^,^51^,^52^,^53^,^54^,^55^,^56^,^57^,^58^,^59^,^60^,^61^,^62^. Here we propose that some of these models can also support another important function namely reflexive spatial attention. We have studied a very simple model but principles from our model may be applied to a network with a larger number of neurons as long as the network is organized with sub-networks following the same architecture as our model. Because, mutual inhibition and repetition suppression are commonly found in many brain areas, our model is consistent with previous proposals of a distributed system in which each module or cortical area can perform reflexive memory and attention functions with varying featural, spatial, and temporal selectivities^41^,^63^,^67^,^84^.

Many elaborate and comprehensive theoretical models have tried to account for and explain the interaction or lack of interaction between attention and working memory (for review see^1^). The nature of the relationship between attention and memory is not well understood. This situation may be in part due to the large and varied cornucopia of behavioral effects attributed to various attentional and mnemonic processes. Our approach differs in several respects. First, our model is focused, restricted, and grounded in neurophysiologically plausible model neurons. We show that a model that can explain reflexive spatial attentional effects also demonstrates maintained activity, such activity often proposed and needed for various memory mechanisms. We argue that such persistent activity may represent the neural correlate of a passive memory trace that may account for aspects of priming, proactive interference and masking. Second, this mechanism is widespread in the brain, neurophysiologically plausible, and does not require implementation in a unique network of brain areas, master saliency map, or memory store as proposed by models of attention (e.g. see^85^). We suggest instead a mechanism that is pervasive and argue that such reflexive attentional effects and passive memory traces are specific and local to the areas and neurons that are responding.

### Model Predictions

One prediction of the model is that behaviorally measured facilitation in reflexive attention paradigms will be affected differently to changes in local inhibition compared to neuronal maintained activity and behaviorally measured passive memory. Based on the model, if local inhibition is decreased (e.g. by blocking GABA receptors on excitatory cells or silencing inhibitory cells), one would expect reduction in maintained activity and behaviorally measured passive memory but increase in reflexive attention (i.e. increased facilitation in reflexive attention paradigms). In addition, decrease in local inhibition would decrease the negative cueing effect in reflexive attention paradigms. Because repetition suppression in inhibitory cells is critical for operation regime, the model would predict qualitative differences in maintained activity of excitatory cells (e.g. change in operating regime) when different types of interneurons are blocked. For example, interneurons expressing somatostatin and vasoactive intestinal peptide exhibit spike rate adaptation^86^. Blocking these interneurons compared to other non-adapting ones should qualitatively change the maintained activity in excitatory cells. In areas where cells exhibit maintained activity (e.g. LIP), the presence or duration of maintained activity (see e.g., task dependent cells in^29^) should depend on inhibition from areas controlling voluntary functions (e.g. perhaps PFC projections). If inhibition projecting cells (can be di-synaptic excitatory neurons) are silenced in voluntary areas, then cells in areas where the blocked inhibition projects should exhibit longer durations of maintained activity. The model also predicts an inverse relationship between baseline activity and maintained activity for excitatory cells operating in the single equilibrium regime. That is, cells with higher baseline firing rates should demonstrate proportionally lower elevation in their delay activity.

In sum, we provide a straightforward 4-cell model circuit that provides a first simple and unified physiologically plausible mechanism of reflexive spatial attention and passive short-term memory processes. We demonstrate that different parameters of the model differentially influence attentional and memory effects, suggesting (1) important tests in physiology and behavior, (2) a distributed and local mechanism for reflexive attention and memory effects, (3) possible avenues to dissociate or discriminate between attention and memory deficits in clinical populations, as well as (4) providing a physiologically plausible mechanism to better develop, define and constrain interventions. A simple physiologically plausible and unified model of attention and memory opens a new frontier that promises to change our understanding of attention and memory.

## Modeling Methods

The modeling methods are similar to those we have published previously (Patel *et al*., 2010). Briefly, we developed a simple model using model neurons with shunting dynamics^87^. We set the parameters to mimic the repetition suppression property of individual neurons in area LIP and included the property of mutual inhibition among these neurons (see Fig. 1A).

In the original model of reflexive spatial attention^67^, there were two spatial locations, but in this paper we only consider one spatial location. The spatial location ‘s’ is normally encoded by a pool of shape selective neurons. For simplicity, we used two shape selective neurons that were selective for shape “a” or “b” (N_a,s_ & N_b,s_). In order to qualitatively mimic the firing profile of a shape selective neuron in area LIP^30^, one key requirement of a shape selective model neuron was adaptive gain control. The adaptive gain control reduced the effectiveness of a stimulus when presented repeatedly by reducing the output of the model neuron, a property referred to as repetition suppression. We do not know if the repetition suppression property observed in LIP neurons is due to biophysical properties of LIP neurons (as we have implemented with our adaptive gain component) or due to a suppressed input to the LIP neuron. Therefore we do not claim that the implementation we have chosen to functionally mimic repetition suppression is exactly how it is implemented in the brain. We have however chosen to utilize a biophysical mechanism for repetition suppression that has been previously used to implement response adaptation in retinal computations^87^,^88^,^89^.

Each shape selective neuron mutually inhibited the other local shape selective neuron via an inter-neuron (IN_ab,s_ & IN_ba,s_). There is indirect evidence of local inhibitory interactions among texture selective neurons in inferotemporal cortex of monkeys^90^. Wang *et al*. showed that blocking GABAergic inhibition in inferotemporal cortex caused previously unresponsive cells to respond to textured stimuli. In other words, removal of inhibition broadened the texture selectivity of the investigated cells. The model was simulated using Matlab (The MathWorks, Natick, MA). The differential equations governing the dynamics of all the neurons in the model and the parameters of the model are described in the supplementary material (see Mathematical Description of the Model section and Table S1, respectively).

## Additional Information

**How to cite this article**: Patel, S. S. *et al*. Single Canonical Model of Reflexive Memory and Spatial Attention. *Sci. Rep*. **5**, 15604; doi: 10.1038/srep15604 (2015).

## Supplementary Material

Supplementary Information
