# Supplementary material for: Single Canonical Model of Reflexive Memory and Spatial Attention
Source: Sci Rep. 2015 Oct 23;5:15604. doi: 10.1038/srep15604 (PMC4616065; doi:10.1038/srep15604)
Supplement: Supplementary Information [file srep15604-s1.pdf]

Supplementary Material

Figure Legend

Figure S1. Relationship between short (light gray) and longer term (dark gray) maintained activity and stimulus pulse amplitude when the model is operating in the dual equilibrium regime. The longer term maintained activity (measured between 10 and 10.35 second interval after 200 msec stimulus pulse, to confirm steady-state) corresponds to the stable equilibrium point of the regime and is independent of the pulse amplitude (from 0.1 to 10 simulation units).

Figure S2. Simulation using model in Patel et al [1] of spatial cueing effect in a reflexive attention experiment described by Posner [2]. The two parameters that differ across panels are W_{inh} and R representing the strength of mutual inhibition and baseline activity, respectively. **A.** Simulated reflexive spatial cueing effects as a function of cue to target asynchrony obtained using the model parameters described in Patel et al [1] (W_{inh}=1, R=0.15). **B.** Simulated reflexive spatial cueing effects obtained when baseline activity (R) was increased to 1. **C.** Simulated reflexive spatial cueing effects obtained when mutual inhibition (W_{inh}) was decreased to 0.55. **D.** Simulated reflexive spatial cueing effects obtained using the model parameters described in this paper (W_{inh}=0.55, R=1).

Figure S3. Relationship of model neuron's response to that of a biological neuron. **A.** Comparison of responses of a shape selective model neuron and, shape selective neuron in monkey LIP shown in Lehky and Sereno [3]. The model simulation was performed with the same parameters listed in Table 1 in the appendix with one exception – the excitatory input representing the stimulus (I_{exc} : on) was set to 4 instead of 10. Similar to the average stimulus

duration in Lehky and Sereno [3], the duration of stimulus in this simulation was set to 250 msec. **B.** Response of the shape selective model neuron obtained using the model parameters used in the study of reflexive spatial attention [1]. Note the presence of activity that is elevated relative to the background. Two model parameters (shown in bold in Table 1 in appendix) are adjusted in this paper to highlight this maintained activity.

Mathematical Description of the Model

The model was simulated using Matlab Simulink (The MathWorks, Inc., MA).

<u>Equations of shape selective neuron's (designated as x) activity dynamics</u>

Dynamics of membrane activity (x) of the jth shape selective neuron:

$$\frac{dx_j}{dt} = -A_x x_j + (B_x - x_j)(I_{e_j} + R) - (x_j - D_x)I_{i_j} + (rand - 0.5)N_{exc} \text{ where, } j \in \{a,b\}$$

$rand$ is a number picked from a uniform distribution between 0 and 1 on each ODE solver iteration

Firing rate (FR) of jth shape selective neuron:

$$FR_{x,j} = \sigma_x f(x_j - \theta)$$

$$f(p) = \begin{cases} p, & p > 0 \\ 0, & otherwise \end{cases}$$

Net excitatory (I_e) and inhibitory (I_i) input to the jth shape selective cell:

$$I_{e_j} = G[\eta_{exc}(I_{exc_j} + \delta I_{exc_k})]\omega_{x,exc}(I_{exc_j} + \delta I_{exc_k}) \; where, \begin{cases} if\, j = a, \; k = b \\ if\, j = b, \; k = a \end{cases}$$

$$I_{i_j} = G[\eta_{inh} I_{inh_j}]\omega_{x,inh} I_{inh_j}$$

$$I_{inh_j} = FR_{y,kj} \; where, \begin{cases} if\, j = a, \; k = b \\ if\, j = b, \; k = a \end{cases}$$

Adaptive gain function (G) in a synapse of the jth shape selective cell, where z and z_0 are the dynamic and baseline gain levels in the synapse:

$$G[p] = z + z_0$$

$$\frac{1}{\tau}\frac{dz}{dt} = \alpha(\beta - z - z_0) - (J + p)\gamma(z + z_0)$$

$$z_0 = \frac{\alpha\beta}{\gamma J + \alpha}$$

<u>Equations of activity dynamics of an inhibitory neuron (designated by y)</u>

Dynamics of membrane activity (y) of the jk^{th} inhibitory inter-neuron (i.e. inhibition from j^{th} neuron to k^{th} neuron):

$$\frac{dy_{jk}}{dt} = -A_y y_{jk} + (B_y - y_{jk})\omega_{y,exc} FR_{x,j} + (rand - 0.5)N_{inh}$$

Firing rate (FR) of jk^{th} inhibitory inter-neuron:

$$FR_{y,jk} = \sigma_y f(y_{jk} - \theta)$$

Table S1: Model Parameters

Description	Label	Value
Passive decay constant of Shape Selective Cell (SSC)	A_x	5
Upper bound of excitatory membrane activity for SSC	B_x	1
Lower bound of inhibitory membrane activity for SSC	D_x	-1
Excitatory synaptic gain for SSC	$\omega_{x,exc}$	1
Inhibitory synaptic gain for SSC	$\omega_{x,inh}$	**0.55** (1)
Firing membrane activity threshold for SSC	θ	0
Membrane potential to firing rate transformation constant for SSC	σ_x	10
Excitatory cross talk between SSCs due to overlapping selectivity	δ	**0** (0.1)
Rate of gain increase in the synapse of SSC	α	0.9
Maximum gain level in the synapse of SSC	β	1
Relative time scale of the gain modulation dynamics in SSC	τ	1
Baseline input adding a tonic gain level in SSC	J	0
Rate of gain decrease in the synapse of SSC	γ	0.1
Scale factor for excitatory synaptic input in SSC	η_{exc}	20
Scale factor for inhibitory synaptic input in SSC	η_{inh}	1
Minimum baseline (or tonic) excitatory synaptic input in SSC	R	**1** (0.15)
Passive decay constant of Inhibitory Inter-Neuron (IIN)	A_y	2
Upper bound of excitatory membrane activity for IIN	B_y	1
Excitatory synaptic gain for IIN	$\omega_{y,exc}$	1
Membrane potential to firing rate transformation constant for IIN	σ_y	5
On state level of external excitatory input signal to SSC (pulse on)	I_{exc} : on	10
*In Fig. 1B, bottom panel	I_{exc} : on	0.5 to 10
Off state level of external excitatory input signal to SSC (pulse off)	I_{exc} : off	0
Noise in membrane activity rate of SSC	N_{exc}	1
Noise in membrane activity rate of IIN	N_{inh}	1

References

1 Patel, S. S., Peng, X. & Sereno, A. B. Shape effects on reflexive spatial selective attention and a plausible neurophysiological model. *Vision Res* 50, 1235-1248, doi:S0042-6989(10)00181-1 (2010).

2 Posner, M. I. Orienting of attention. *Q J Exp Psychol* 32, 3-25 (1980).

3 Lehky, S. R. & Sereno, A. B. Comparison of shape encoding in primate dorsal and ventral visual pathways. *J Neurophysiol* 97, 307-319, doi:00168.2006 (2007).

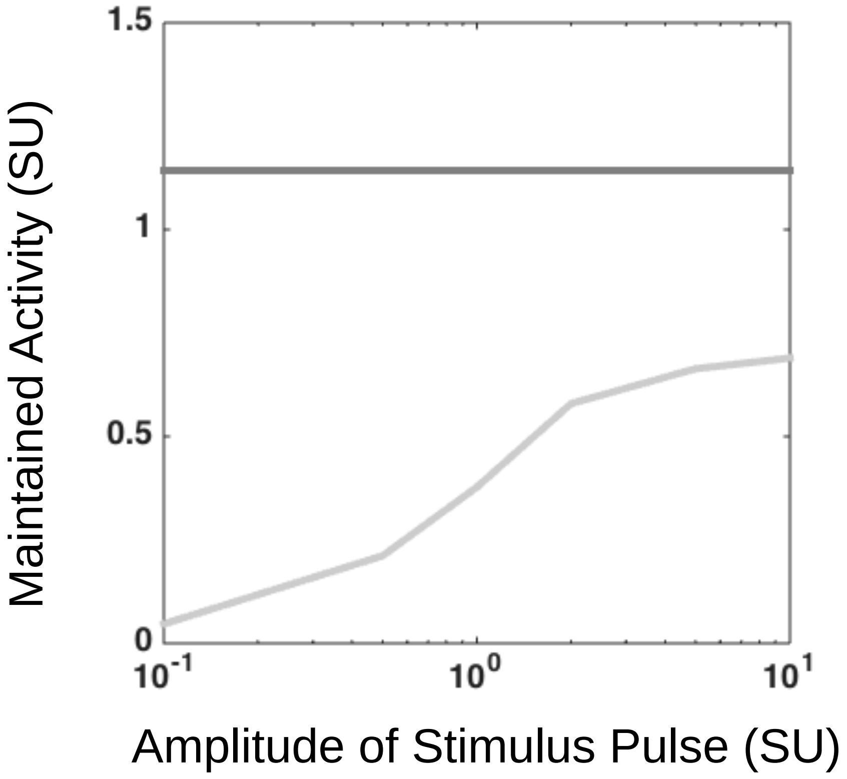
1.5
1
0.5
0
10^{-1}
10^{0}
10^{1}
Maintained Activity (SU)
Amplitude of Stimulus Pulse (SU)

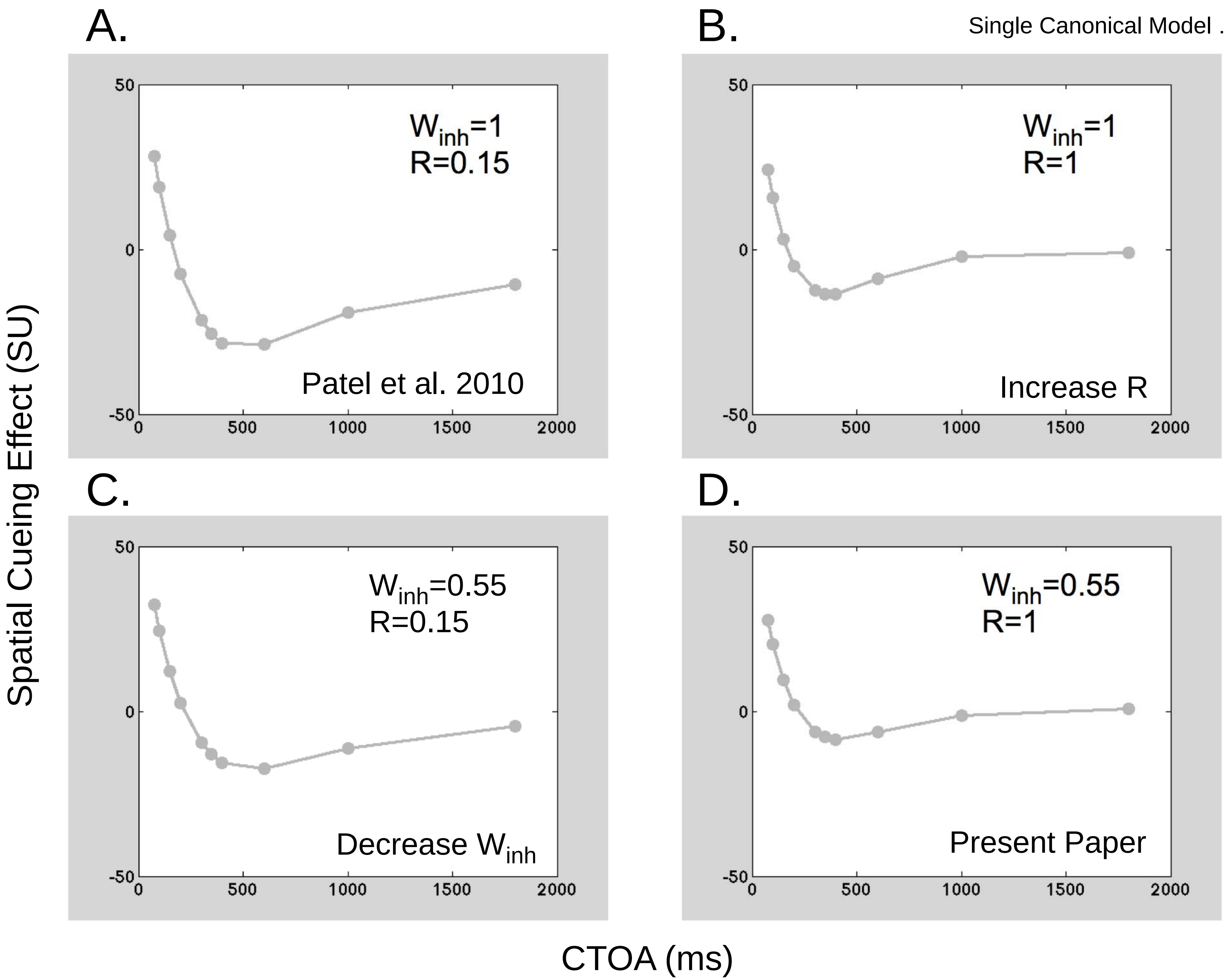
A.
B.
C.
D.
Spatial Cueing Effect (SU)
CTOA (ms)
W$_{inh}$=1
R=0.15
Patel et al. 2010
W$_{inh}$=1
R=1
Increase R
W$_{inh}$=0.55
R=0.15
Decrease W$_{inh}$
W$_{inh}$=0.55
R=1
Present Paper
50
0
-50
0
500
1000
1500
2000

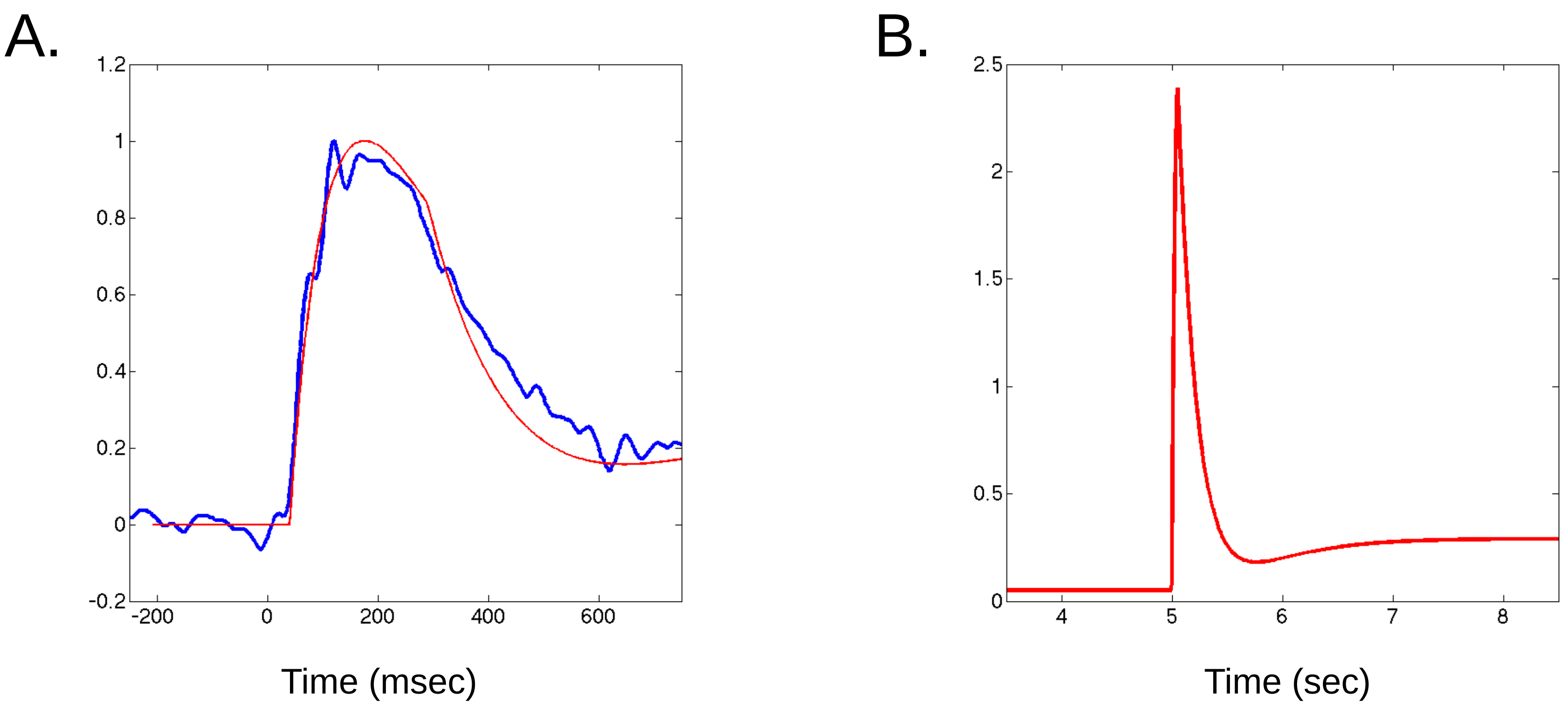
A.
1.2
1
0.8
0.6
0.4
0.2
0
-0.2
-200
0
200
400
600
Time (msec)
B.
2.5
2
1.5
1
0.5
0
4
5
6
7
8
Time (sec)
